# Appropriate and mindful measurement of serum calcitonin in patients with thyroid nodules. A white paper

**DOI:** 10.1007/s12020-023-03485-6

**Published:** 2023-08-17

**Authors:** Pierpaolo Trimboli, Pablo Valderrabano, Fabian Pitoia, Arnoldo Piccardo, Jörg Bojunga

**Affiliations:** 1 Servizio di Endocrinologia e Diabetologia, Ente Ospedaliero Cantonale (EOC), Lugano, Switzerland; 2 Facoltà di Scienze Biomediche, Università della Svizzera Italiana (USI), Lugano, Switzerland; 3 Department of Endocrinology and Nutrition, Hospital Universitario Ramón y Cajal, IRYCIS, Madrid, Spain; 4 Hospital de Clínicas José de San Martín—University of Buenos Aires, Buenos Aires, Argentina; 5 Department of Nuclear Medicine, E.O. “Ospedali Galliera”, Genoa, Italy; 6 Department of Internal Medicine 1, Goethe University Hospital Frankfurt, Goethe-University, Frankfurt am Main, Germany

**Keywords:** Calcitonin, Thyroid nodule, Medullary thyroid carcinoma

## Abstract

Medullary thyroid carcinoma (MTC) is an infrequent thyroid malignancy that is often diagnosed at advanced stage with consequent poor prognosis. Thus, the earlier the diagnosis of MTC, the better the prognosis. Unfortunately, the preoperative detection of MTC remains challenging in clinical practice. In fact, while ultrasound and fine-needle aspiration cytology have suboptimal performance in this context, measuring serum calcitonin (Ctn), fully recognized as the most reliable test to detect MTC, is not universally accepted as routine test in all patients with thyroid nodule(s). The authors of this paper reappraise critically the matter of Ctn measurement in view of the recent advancements in the literature to point out the essential information to be known, and then to prepare an easy-to-use guide for clinicians to appropriately consider the measurement of serum Ctn during clinical practice.

## Background

Medullary thyroid carcinoma (MTC) is an infrequent malignant tumor originating from thyroid C cells. It was firstly described in 1951 [1] and the presence of “thyrocalcitonin” was later explored [2]. One in four cases is inherited as familial MTC or within multiple endocrine neoplasia (MEN) type 2 A or 2B syndromes [3]. MTC is often diagnosed with more advanced stage than well-differentiated thyroid cancer and is associated with poorer prognosis. Thus, the earlier the diagnosis of MTC, the better the prognosis [3]. The early spread of MTC to regional lymph nodes makes generally necessary to treat MTCs with total thyroidectomy and lymph node dissection, of at least the central compartment. In fact, an incomplete initial resection significantly decreases the chance to achieve structural and biochemical complete response. Furthermore, patients diagnosed with advanced MTC can be treated with systemic therapies with only a minority of tumors –about 10% of *RET*-mutated tumors– achieving complete response with selective *RET* inhibitors; while the other 90% can expect a transitory partial response at best and a median progression free survival of 2–3 years with multikinase inhibitors and (probably) longer with selective RET inhibitors [4]. Thus, early presurgical diagnosis of MTC has a direct impact on treatment’s outcomes and patient’s prognosis.

Unfortunately, the preoperative detection of MTC remains challenging in clinical practice [5]. Most MTCs are solid and hypoechoic at ultrasound (US) and often have macrocalcifications [6]. However, suspicious US features are less frequent in MTC than in papillary thyroid carcinoma, even if their prevalence among MTCs is higher than among follicular carcinomas. This makes sense, given that suspicious sonographic features were derived from cohorts in which >90% of the malignancies were papillary thyroid carcinomas [7]. Furthermore, no sonographic features are specific of MTC. Therefore, depending on the sonographic classification used to set the threshold for biopsy, a significant proportion of MTCs may not be eligible for biopsy. Thus, delaying diagnosis with possible prognostic implications. If fine-needle aspiration cytology (FNAC) is performed, only about 50–60% of MTC cytological specimens allow a direct MTC diagnosis [8]. The remainder of specimens are either non-diagnostic or classified into an indeterminate category of thyroid cytopathology; thus, being subject to either follow-up or diagnostic surgery, both of which can be detrimental to patient’s prognosis. In this context, while the measurement of serum calcitonin (Ctn) is fully recognized as the most reliable test to detect MTC [9], its routine use in all patients with thyroid nodule(s) has not been universally accepted.

## Recommendations from International guidelines about serum Ctn measurement

The 2006 consensus of European Thyroid Association (ETA) was in favor of routine serum Ctn testing during the initial assessment of patients diagnosed with thyroid nodule [10]. However, this recommendation was modified in the 2010 joint guidelines from ETA, American Association of Clinical Endocrinologists (AACE), and Associazione Medici Endocrinologi (AME) which recommended serum Ctn measurement for specific patient settings only, i.e., in the presence of relatives diagnosed with MTC and before surgery to avoid difficult follow-up in case of incidental histological diagnosis of MTC [11]. The 2015 American Thyroid Association (ATA) guidelines for the management of patients with thyroid nodules and differentiated thyroid cancer were neither in favor nor against routine serum Ctn measurement [12]. Similarly, the 2015 MTC ATA guidelines included the following recommendation: “*Realizing that opinions of experts vary regarding the usefulness of measuring serum Ctn levels in patients with nodular goiters, the Task Force recommends that physicians decide whether the technique is useful in the management of patients in their clinic*” [3]. More recently, the 2023 ETA guidelines for thyroid nodule management recommends measuring Ctn in specific scenarios, such as in patients scheduled for surgery or thermal-ablation, or with nodules with indeterminate cytology or suspicious sonographic pattern, or with family history of MTC or MEN. [13].

## Purpose of the paper

The lack of clarity on whether serum Ctn should be tested and in which circumstances prompted the authors of this white paper to critically reappraise the matter, considering recent advancements in this field. This document is then intended to be an easy-to-use guide for clinicians to appropriately consider the measurement of serum Ctn during the evaluation of their patients with thyroid nodules.

## Essentials

1. MTC is a malignant cancer with early spread to regional lymph nodes and frequent distant metastases (about 10%) at diagnosis [3]. Thus, early its detection improves patient prognosis and treatment outcomes.
2. US has suboptimal performance in identifying MTC and only about a half of cases are classified as high-risk nodules according to current risk stratification systems. It is unknown how many MTC can be eligible for FNAC according to the size thresholds included in risk stratification systems. Subcentimetric MTCs can resemble papillary microcarcinomas on ultrasound [6, 14].
3. FNAC can correctly detect MTC in only 50–60% of cases [8, 15]. Some specimens from MTC can be interpreted as benign and some other MTCs can be misdiagnosed due to the presence of oncocytic changes [16].
4. Serum Ctn is the most reliable marker for MTC diagnosis [9].
  - Serum Ctn values > 100 pg/mL are very specific for MTC with negligible false-positive rates [3].
  - Serum Ctn levels <10 ug/mL exclude, in general, MTC diagnosis, with the rare exception of non-secretory MTCs [11, 12, 17].
  - Mild serum Ctn increase may be due to interfering factors, non-thyroidal pathological conditions, or pure determinants, such as thyroid size, thyroid autoimmunity, presence of chronic kidney disease, bacterial infection, use of proton pump inhibitors, smoking, and patient’s gender, age, and anthropometrics [3]. It should be taken into account that other non-thyroid malignancies (i.e., neuroendocrine neoplasia) can produce Ctn [18].
  - The rate of patients with indeterminate serum Ctn values, between 10 and 100 pg/mL, is negligible (1.7%); however, the prevalence of MTC in this setting is close to 10%, increasing significantly above 20 pg/mL [19]. This is the reason why some experts adopted in clinical practice different cut-offs to differentiate micro-MTC from C-cell hyperplasia and other unspecific Ctn elevations, such as 30 pg/mL in females and 60 pg/mL in males [20].
  - Unfortunately, there is no consensus about fixed cut-off of basal Cnt to be used in clinical practice. This is mainly due to the wide heterogeneity among studies enrolling series with different MTC prevalence [19, 21–24].
  - Ctn levels above 500 pg/mL preoperatively and/or above 150 pg/mL postoperatively are suspicious for metastatic MTC, making necessary further imaging to rule out disease outside the neck [3, 9].
  - The prevalence of MTC in populations with thyroid nodule(s) undergoing routine serum Ctn measurement ranges between 0.11% and 0.85% with a median of 0.32% [23, 24].

## Advantages of routine serum Ctn testing in patients with thyroid nodule(s)

1. Ctn levels >100 pg/mL are very specific for MTC. Given the prevalence of *RET* germline mutations, pheochromocytoma must be ruled out preoperatively in all patients diagnosed with MTC. Furthermore, serum Ctn >500 pg/mL are suspicious for distant spread and require further imaging that can change treatment recommendations and surgical extent [3].
2. Even if thyroid US and FNAC are the pivotal tests for the evaluation of patients with thyroid nodules, MTC cannot be excluded in cases with non-suspicious/indeterminate sonographic or cytologic findings. Routine use of serum Ctn allows to overcome these relevant limitations for MTC diagnosis. Furthermore, in the presence of elevated pre-FNAC serum Ctn, FNAC can be combined with the measurement of Ctn in the needle washout and with Ctn immunocytochemistry to improve its diagnostic performance. Pathologists can also improve their diagnostic performance on cytological specimens being aware of the biochemical suspicion for MTC [15].
3. Although infrequent, some MTCs may not secrete Ctn. The postoperative follow-up of these tumors, usually diagnosed upon histological examination, can be misled by negative post-surgical serum Ctn levels. Having a negative presurgical serum Ctn level, however, prevents this rare pitfall from happening [17].

## Implications of routine serum Ctn testing in patients with thyroid nodule(s)

1. Ctn levels >100 pg/mL confirm MTC and levels <10 pg/mL exclude it, with insignificant false negative and false positive rates. However, there is a wide range of indeterminate serum Ctn levels [3]. These levels may be due to small MTC, C cell hyperplasia (considered a pre-malignant lesion), interfering factors, or individual characteristics. The clinical management of these cases can be challenging. The prevalence of indeterminate serum Ctn levels, however, is very low (<2%); whereas MTC prevalence is not negligible in this setting (around 10%), and increases with increasing serum Ctn levels [19].
  Once interfering factors are ruled out, further diagnostic work-up can be discussed with both patient and surgeon considering the following issues:
  - Serum Ctn levels are associated with the probability of MTC [3];
  - Serum Ctn levels correlate, in general, with the tumor burden. Thus, if MTC is present with indeterminate Ctn levels, it is likely a small, low-stage MTC [3];
  - The diagnostic work-up can be extended with further evaluation. FNAC can be combined with measurement of Ctn in the needle washout. The technique demonstrated significantly higher sensitivity in detecting MTC than cytology in a head-to-head comparison [15]. Serum Ctn stimulation with calcium injection was reported as easy-to-use with improvement of sensitivity with respect basal Ctn [21]. Anyway, both Ctn measurement in needle washout and calcium-stimulated serum Ctn have not fixed thresholds to be used and require to develop an institutional cut-off [15, 21, 22]. In addition to these two major tools in this context, immunocytochemistry examination for Ctn can be performed on cytological smears [25], and other serum markers can be tested to refine the risk of MTC [26, 27].
2. The role of imaging procedures other than neck US in low-stage MTC has not been fully explored [9]. Therefore, their use cannot be recommended in patients with mild Ctn increase.
3. The rate of MTC among consecutive patients with thyroid nodules is low (0.3%) [23, 24]. However, it is unknown at this time how many of these MTCs do not meet the biopsy threshold of their specific ultrasound pattern of suspicion, although it is expected to be a fraction of these nodules. If we were to estimate that 30% of MTCs present in a subclinical stage (size below the suspicion pattern-specific threshold for biopsy), routine serum Ctn testing would identify 0.9 cases every 1000 patients with thyroid nodules tested. Therefore, the cost of universal serum Ctn screening can be important and merits attention. The cost and reimbursement criteria for serum Ctn varies significantly across countries; thus, cost-effectiveness needs to be further individualized [28].
4. Several assays of serum Ctn are commercialized with different technical performances and reference ranges. This should be considered when facing patients with mild serum Ctn increase. The possibility of “macrocalcitonin” has to be considered as a potential pitfall for mild-to-moderate serum Ctn levels [29].

## Scenarios in which selective serum Ctn testing might be appropriate to avoid MTC misdiagnosis/delayed diagnosis

Routine serum Ctn measurement might not be cost-effective in many countries in unselected patients with thyroid nodules. However, serum Ctn might be helpful in selected scenarios with an expected higher prevalence of MTC:

1. Family history of MTC or MTC-associated syndrome. In these cases, Ctn measurement is mandatory.
2. Patients undergoing surgery for nodular goiter. Measuring serum Ctn before surgery can be useful to uncover MTC, which can modify the surgical extent and improve chances for biochemical cure.
3. Patients undergoing FNAC. Having indeterminate/high serum Ctn levels allows preparing a tube for Ctn measurement in the needle washout; and provides useful information for pathologists’ cytological evaluation, possibly reducing the chance to miss a diagnosis.
4. Patients with sub-centimetric thyroid nodules with high-suspicion US pattern (equivalent to ACR or EU-TIRADS category 5). According to some guidelines, these nodules could be followed up and not biopsied unless there is suspicion for extrathyroidal extension or presence of regional or distant metastases. However, small MTCs may look like very low-risk papillary microcarcinomas. Thus, ruling out MTC before embarking on active surveillance seems sensible. Serum Ctn might be within the indeterminate range in this situation given the small size of the primary tumor.
5. Patients with biopsied thyroid nodules in which the pathologist suggests ruling out MTC. If serum Ctn is within the indeterminate range, repeating FNAC with Ctn measurement in the needle washout might provide a definitive diagnosis.
6. Thyroid nodules with indeterminate cytology (Bethesda III or IV, or equivalent categories of other thyroid cytology classification systems), particularly in aspirates with oncocytic changes. The presence of follicular cells with architectural atypia and presence of colloid probably makes serum Ctn measurement unnecessary.
7. Thyroid nodules with repeated non-diagnostic cytology (Bethesda I or equivalent categories of other thyroid cytology classification systems). These nodules might be offered US follow-up or diagnostic surgery, both of which would be inappropriate for MTC.

## Conclusions for clinical practice

MTC is rare but needs to be considered routinely when evaluating patients with thyroid nodules, as delayed diagnosis can have significant prognostic implications. Thyroid ultrasound and cytology have limited ability to identify a significant proportion of MTCs. Appropriate Ctn measurement in patients with thyroid nodules might help to overcome these relevant limitations in clinical practice.

## Data Availability

The data sets used and/or analyzed during the current study are available from the corresponding author on reasonable request.
